# Therapeutic engineering of the gut microbiome using synthetic biology and metabolic tools: a comprehensive review with *E. coli* Nissle 1917 as a model case study

**DOI:** 10.1007/s00203-025-04417-w

**Published:** 2025-08-06

**Authors:** Soumok Sadhu, Tania Paul, Nishant Yadav

**Affiliations:** 1 Department of Veterinary Sciences and Biomedical Sciences, College of Veterinary Medicine, University of Minnesota, Minneapolis, USA; 2 Department of Medicine, Medical School, University of Minnesota, Minneapolis, USA; 3 Department of Microbiology, Swami Vivekananda Institute of Modern Sciences, Rajpur Sonarpur, West Bengal India; 4 Department of Civil Engineering, SoS, E&T, GGV, Bilaspur, India

**Keywords:** Gut microbiome, Metabolic engineering, *Escherichia coli* Nissle 1917, Fecal microbiota transplantation, Short-chain fatty acids

## Abstract

The human gut microbiome significantly influences host physiology, metabolism, and immune function. The engineering of microbial communities represents a significant advancement in contemporary biotechnology. Conventional methods, including Fecal Microbiota Transplantation (FMT) and probiotic administration, exhibit limitations in efficacy and raise safety and reproducibility concerns; however, they have shown potential therapeutic benefits. Recent progress in biocatalysis and metabolic engineering has led to the development of genetically tractable gut bacteria for targeted therapeutic purposes, particularly in the last five years. This chapter offers an overview of the development of microbiota-based interventions, from early recombinant probiotics to advanced synthetic biology platforms that can detect and respond to host and environmental signals. This analysis examines the mechanistic aspects of enzyme engineering, including improvements in metabolic pathways for the production of short-chain fatty acids, the breakdown of harmful metabolites, and the biosynthesis of immunomodulatory compounds. This review also examines conditions including inflammatory bowel disease, metabolic dysfunction, and colorectal cancer, highlighting microbial production systems pertinent to gut health. The engineering of *Escherichia coli* Nissle 1917 to produce phenylalanine ammonia-lyase (PAL) and L-amino acid deaminase (LAAD) represents a significant advancement in gut-based metabolic intervention for patients with phenylketonuria (PKU) by degrading excess phenylalanine. Recent studies offer peer-reviewed evidence supporting the translational potential of these inventions, as demonstrated through figures and tables highlighting engineered metabolic circuits, therapeutic outputs, and strain performance metrics. This combination of developments demonstrates the potential of synthetic microbiome engineering to provide precision biotherapeutics for various gut-related conditions.

## Introduction

The gut microbiota is a complex environment that has a big effect on the host’s metabolism and immunity. Dysbiosis, characterized by imbalances in the gut microbiome, has been associated with conditions such as obesity, diabetes, inflammatory bowel disease (IBD), and neurobehavioral disorders (Durack and Lynch 2019). Microbiota-based therapies are receiving more attention as efforts to rectify dysbiosis and improve gut health have intensified. Two initial treatments that showed potential are the administration of probiotic strains and Fecal Microbiota Transplantation (FMT), which involves transferring entire microbial communities from healthy donors. Probiotic bacteria inherently contribute to human health by improving nutrition and preventing infections (Kumari et al. 2022), providing a strong foundation for developing engineered gut microbes with enhanced therapeutic functions. FMT may induce adverse effects (e.g., gastrointestinal disturbances, fever) under some circumstances, and its mechanisms of action remain only partially understood. Conventional probiotic therapy, including those utilizing lactic acid bacteria, frequently yield inconsistent therapeutic results and may occasionally result in adverse effects (Biazzo and Deidda 2022). These limitations have prompted researchers to seek more precise and manageable methodologies.

**Fig. 1:**
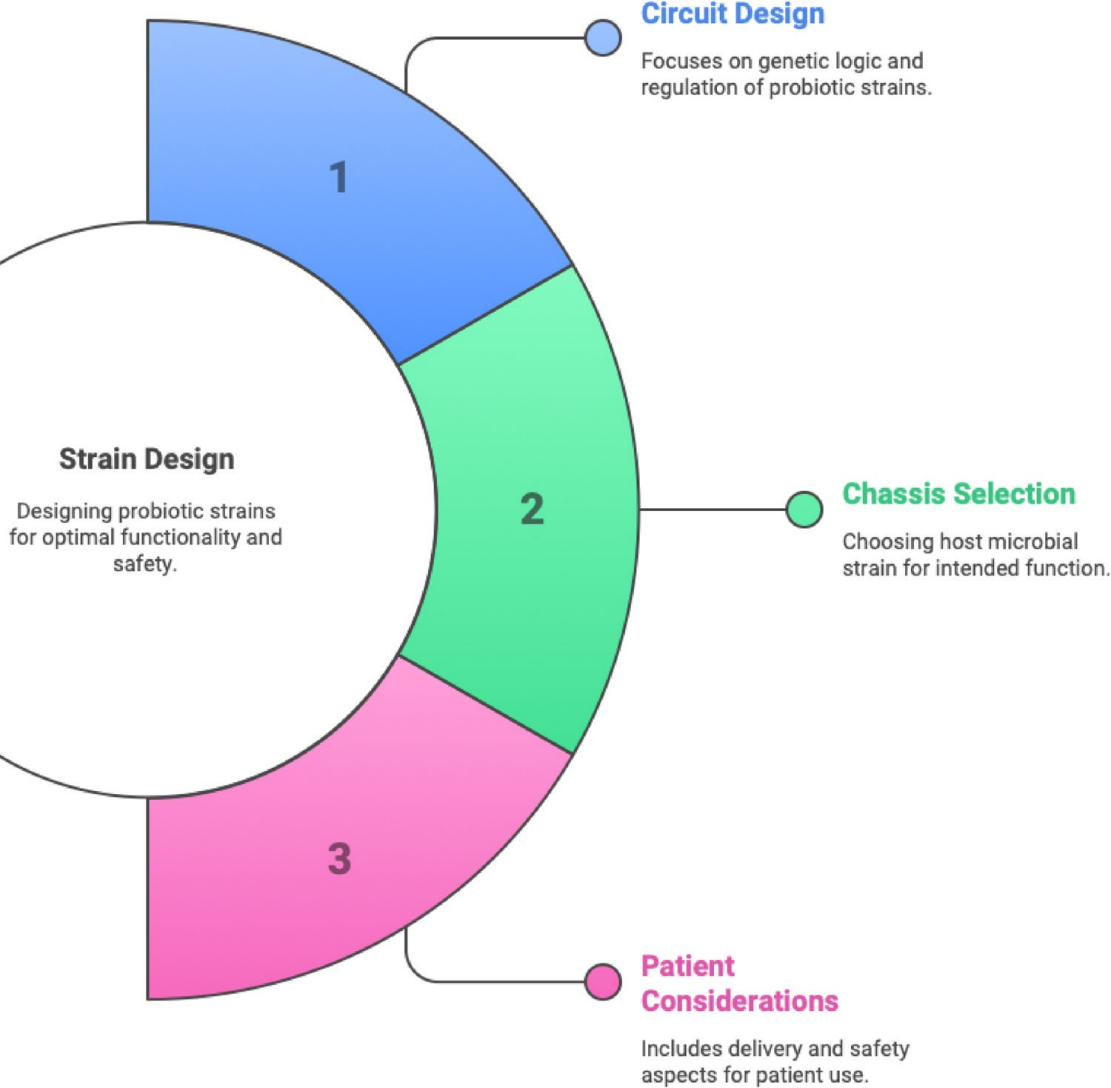
Key parameters for probiotic strain design. Strain design for engineered live bacterial therapeutics involves a three-pronged approach: (1) Circuit Design, focusing on the genetic logic and regulatory elements to ensure optimal expression and functionality of therapeutic payloads; (2) Chassis Selection, which refers to identifying a suitable host microbial strain capable of surviving and performing in the target environment; and (3) Patient Considerations, including delivery mode, dosing strategy, and safety aspects for clinical feasibility and patient compliance. Balancing these design principles is essential for the development of robust and effective microbial therapeutics. (Adapted from Charbonneau et al. 2020)

Recent advancements in the metabolic engineering of gut bacteria offer a powerful solution to the limitations of Fecal Microbiota Transplantation and conventional probiotics. Metabolic engineering, as stated by (Li et al. 2023), involves the application of engineering principles and genetic techniques to modify the metabolic pathways of organisms. This entails genetically reprogramming bacteria inside the gut microbiome to perform enhanced or novel biochemical functions beneficial to the host. Therapeutic actions beyond the capabilities of the native microbiota can be achieved by endowing bacteria with novel enzymatic activity or regulatory circuits (Charbonneau et al. 2020). Live bacterial therapeutics offer distinct advantages over unmodified microbiota treatments: they can be engineered to execute functions absent in natural gut bacteria, such as rapid detoxification of metabolites or the delivery of human cytokines (Charbonneau et al. 2020). Designing such live biotherapeutics requires careful consideration of three integrated factors: (1) genetic circuit design to control therapeutic output and safety, (2) selection of an appropriate microbial chassis capable of gut colonization, and (3) patient considerations such as dosage, delivery method, and safety. These principles are both described here in text and visually summarized in Fig. 1 to enhance conceptual understanding.

**Fig. 2:**
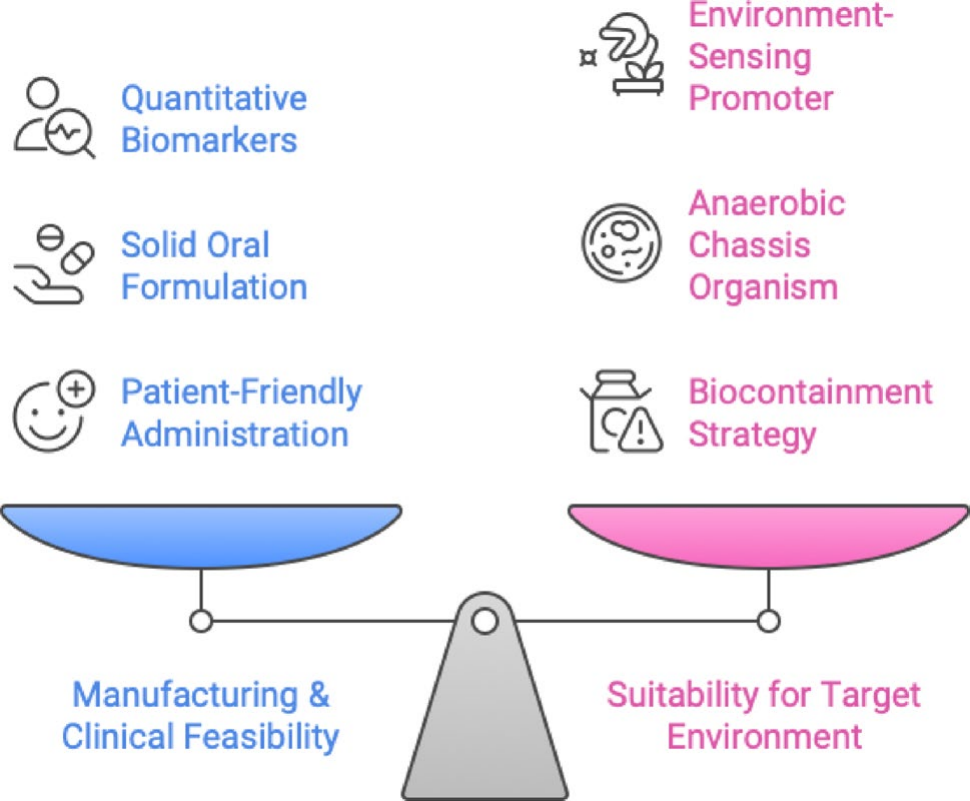
Trade-offs in engineered probiotic development. A balanced approach is required between two competing priorities: Manufacturing and Clinical Feasibility (blue), which includes aspects like quantitative biomarkers, solid oral formulation, and patient-friendly administration; and Suitability for the Target Environment (pink), which requires the integration of features such as environment-sensing promoters, anaerobic chassis organisms, and biocontainment strategies. This trade-off framework illustrates the challenges in optimizing both real-world deployment and microbial function in vivo (colour figure online). (Adapted from Charbonneau et al. 2020)

Recent studies have demonstrated the feasibility of developing synthetic microbial groups and next-generation genetically engineered probiotics to combat various diseases. Innovative research conducted two decades prior offered initial proof-of-concept; for example, a 2022 study engineered *Lactococcus lactis* to secrete human interleukin-10, thereby successfully alleviating colitis in murine models (Ma et al. 2022). This research demonstrated that biotherapeutic molecules can be reengineered from gut microbiota. Recent advancements in DNA synthesis, in vitro evolution, and CRISPR-based genome editing have significantly enhanced the metabolic engineering toolkit. In recent years, synthetic biology techniques, including bespoke promoters, logic gene circuits, and CRISPR/Cas editing systems, have been tailored for human gut commensals, facilitating more advanced changes (Yeh et al. 2024). Contemporary engineered microorganisms can identify gut inflammation and secrete anti-inflammatory compounds, produce missing metabolites in situ, or function as live diagnostics by providing information on gut conditions. To further clarify the practical design constraints in engineering microbial therapeutics, Fig. 2 highlights the inherent trade-offs between product manufacturability and functionality in the gut environment. While many early-stage studies emphasize molecular innovations, translating these into viable therapies requires balancing clinical logistics, such as oral formulation and safety testing with biological performance in the host. This visual serves as a conceptual map that simplifies the complexity of strain design and supports the discussion around the real-world deployment of synthetic microbial systems.

This chapter will first talk about the mechanical basis of biocatalysis in engineered gut bacteria. It will focus on how certain enzyme pathways can be improved or added to produce desired metabolic outputs. We subsequently examine notable technological advancements from enzyme engineering techniques to synthetic biology systems, that have advanced the discipline in recent years. Important applications of gut microbiome engineering are being explored in cancer, infections, inflammatory bowel disease, and metabolic illnesses (e.g., diabetes mellitus (Sarkar et al. 2023)). In light of its significant citation profile and clinical importance, we focus a case study on *Escherichia coli* Nissle 1917 (EcN), a model probiotic organism extensively modified as a therapeutic framework. Finally, we examine the current application of these created microbes, including instances from clinical trials, and contemplate potential challenges in microbiome engineering, such as safety, regulation, and gut ecological stability. While this review focuses on E. coli Nissle 1917 (EcN) as a model case study because it is widely used and has advanced engineering strategies. However, it also includes examples of other gut microbes to show how synthetic microbiome therapeutics work in general.

## Innovations in enzyme engineering and synthetic biology tools

**Fig. 3:**
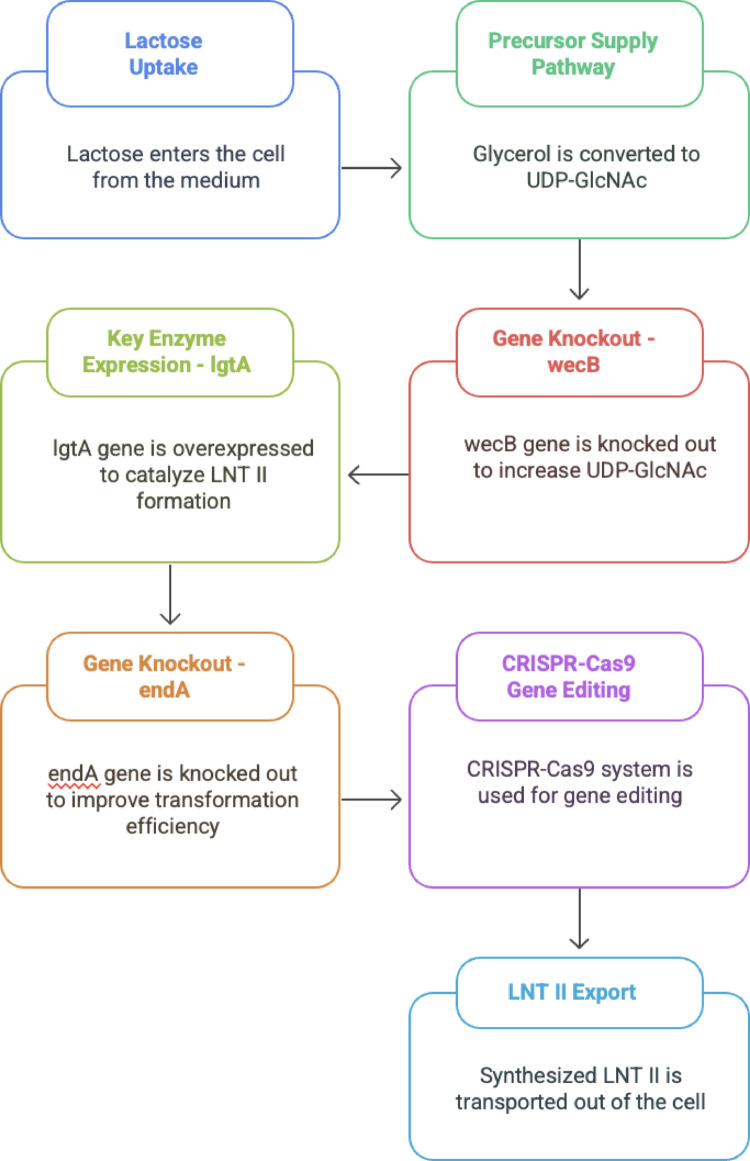
Genetic engineering workflow of* Escherichia coli* Nissle 1917 for Lacto-N-triose II (LNT II) production. This schematic illustrates the rational design and engineering steps employed to enhance LNT II biosynthesis. Key steps include lactose uptake, conversion of glycerol into UDP-GlcNAc precursors, knockout of the wecB gene to reroute UDP-GlcNAc, and overexpression of the lgtA gene encoding β-1,3-N-acetylglucosaminyltransferase. Additionally, endA deletion enhances transformation efficiency. CRISPR-Cas9 is used for precise genome editing. The engineered strain secretes LNT II extracellularly, demonstrating the utility of* E. coli* Nissle 1917 as a programmable biocatalyst for therapeutic oligosaccharide production

The fast advancement in designing gut microbes is motivated by parallel discoveries in synthetic biology and enzyme engineering. The development of genome editing technologies tailored for gut commensals is a notable area of innovation. Historically, numerous gut bacteria, including prominent species such as *Bacteroides* and *Clostridia*, posed challenges for genetic modification. Over the past five years, researchers have successfully utilized CRISPR-Cas systems for precise editing in these species (Yeh et al. 2024). For example, demonstrated a CRISPR/Cas12a-based genome editing method for various human gut Bacteroides species. This toolkit accelerates the construction of modified strains by enabling targeted gene knockouts or insertions to reconfigure metabolism. Ensuring high yields and stability of engineered strains may require systematic optimization of growth conditions, much like the response-surface methodology used to improve mammalian cell culture performance (Sadhu and Sadhu 2023). This progression is exemplified by recent efforts to engineer *E. coli* Nissle 1917 for the biosynthesis of bioactive oligosaccharides such as lacto-N-triose II (LNT II), a key component of human milk oligosaccharides (Fig. 3). In this system, lgtA is overexpressed to catalyze LNT II synthesis from lactose, and precursor flux is enhanced via glycerol-derived UDP-GlcNAc. To increase intracellular UDP-GlcNAc levels and reduce pathway competition, wecB was disrupted, and endA, encoding endonuclease I, was inactivated to improve transformation efficiency. The study employed CRISPR-Cas9 for targeted editing and successfully demonstrated high LNT II yield in bioreactor settings, offering a model workflow for probiotic-based glycan production (Hu and Zhang 2024).

Lacto-N-triose II (LNT II) is a basic building block of human milk oligosaccharides (HMOs) and is the starting point for bigger oligosaccharides like lacto-N-tetraose (LNT) and lacto-N-neotetraose (LNnT) (Fang et al. 2024). This trisaccharide has gotten a lot of attention because it could be a nutraceutical and it helps make more complex HMOs (Zhu et al. 2021). Research shows that LNT II can help good gut microbes grow and improve the health of babies’ guts, similar to some of the benefits of human breast milk. A recent preclinical safety study found that even high doses of LNT II had no negative effects. This supports the idea that it is safe to add to infant formula and functional foods at levels similar to those found in human milk (Fang et al. 2024).

Because of these good qualities, there is a strong desire to use engineered microbes to make LNT II. Metabolic engineering projects, some of which use the probiotic *E. coli* Nissle 1917 (EcN), have gotten very high yields of LNT II. For example, after making several improvements to its pathways, one optimized *E. coli* strain (EcN in a recent study) was able to make 46.2 g/L of LNT II in fed-batch fermentation (Zhu et al. 2021). These improvements included increasing the supply of UDP-GlcNAc, overexpressing the lgtA glycosyltransferase, relieving feedback inhibition (for example, by mutating glmS), and getting rid of pathways that compete with each other (Zhu et al. 2021). The success of this plan shows that synthetically engineered probiotics can be used as effective cell factories for making HMOs. In short, LNT II has become a useful therapeutic and nutritional oligosaccharide as well as a model target that shows how synthetic biology tools can change gut microbes to make health-promoting sugars on a large scale.

In addition to altering native genes, synthetic biologists have created modular genetic elements designed for gut microbes. Included are stable plasmid vectors capable of surviving in the competitive gut environment, anaerobic promoters, and inducible systems operating under low-oxygen circumstances. Yeh and associates developed precisely regulated promoters in *Bacteroides thetaiotaomicron* that respond to food sugars, enabling dietary modulation of therapeutic gene expression (Yeh et al. 2024). Utilizing DNA segment inversion, other teams developed recombinase-based memory circuits in *E. coli* that monitor inflammation by producing a “history” of gastrointestinal inflammatory occurrences discernible from fecal bacteria (Ma et al. 2022). These synthetic circuits are novel instruments enabling modified bacteria to not only produce medication but also to detect, process, and retain signals inside the gastrointestinal environment.

**Fig. 4:**
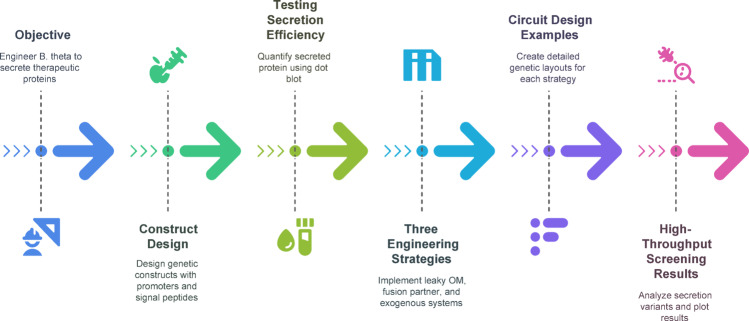
Workflow for engineering therapeutic protein secretion in Bacteroides thetaiotaomicron. This figure summarizes the multi-step strategy used to engineer a gut commensal bacterium for efficient extracellular delivery of a therapeutic protein. The process begins with the overall objective of expressing and secreting a model therapeutic (sdAb-TcdA) using B. thetaiotaomicron as a chassis. Genetic constructs are designed with various signal peptides and promoters to drive expression and export. Secretion efficiency is evaluated using dot blot assays, alongside growth curve analyses to ensure constructs do not impact host viability. Three secretion strategies are implemented: (1) Leaky outer membrane vesicle (OMV)-based release via native pathways, (2) fusion of the therapeutic protein to abundant endogenous membrane proteins, and (3) exogenous secretion systems such as the Type I hemolysin system and Type 8 secretion system from E. coli. Representative genetic circuits illustrate the modular design framework. High-throughput screening of 60 construct variants identifies the most effective secretion configurations based on signal intensity. This pipeline establishes a modular toolkit for programmable protein delivery using live bacterial therapeutics. (Adapted from Yeh et al. 2024)

Facilitating the proper operation of heterologous enzymes in vivo is a significant challenge in enzyme engineering for gastrointestinal applications. Gut bacteria typically must secrete therapeutic proteins, such as anti-inflammatory cytokines or antimicrobial peptides, into the intestinal lumen. Recent investigations have focused on developing secretory pathways to address this issue. A 2024 study by Yeh and associates provided a molecular toolset for protein secretion in *Bacteroides*, identifying signal peptides and secretion pathways that enhance the export of therapeutic proteins. The complete pipeline for engineering and screening secretion systems in *Bacteroides thetaiotaomicron* is illustrated in Fig. 4, showcasing secretion strategies and construct optimization for high-yield therapeutic delivery. Evaluating several native secretion signal sequences in *Bacteroides* enabled the optimization of constructs capable of secreting functional nanobodies and cytokines at elevated quantities (Yeh et al. 2024). Advancements in protein engineering ensure that designed gut microorganisms can directly administer substantial biotherapeutics (e.g., enzymes, antibodies) at the mucosal site of action, a capability that was previously limited.

Directed evolution and computational enzyme design to enhance catalytic efficiency in gastrointestinal circumstances represent another advancement. Enzymes designed for intestinal function must exhibit activity at neutral pH and amidst complex mixtures of metabolites. Researchers have commenced employing directed evolution on enzyme variations within probiotic strains to select mutants exhibiting enhanced activity or stability in fecal-like environments. A phenylalanine ammonia lyase enzyme utilized in a genetically modified *E. coli* was computationally altered to withstand feedback inhibition and enhance its kinetics for the gastrointestinal environment. While much of enzyme engineering thus far has relied on utilizing robust enzymes from nature, such as phenylalanine ammonia lyase from Anabaena or urease from Proteus, there is an emerging trend towards optimizing enzyme performance through rational design, thereby ensuring that engineered pathways operate at peak efficacy in vivo.

Lastly, it is essential to highlight the incorporation of in silico metabolic models. Advanced genome-scale metabolic models (GEMs) of gut bacteria and microbiome populations are currently employed to inform engineering strategies (Esvap and Ulgen 2021). Researchers can predict which gene deletions or additions will steer production towards a desired outcome by simulating the metabolic network of a bacteria. Recent computer modeling demonstrated that eliminating certain fermentation pathways in *E. coli* would redirect carbon towards increased butyrate synthesis; this informed the creation of a strain with knockouts in lactate and succinate production pathways, thereby enhancing butyrate yield experimentally (Ciocan and Elinav 2023). Microbiome research has been refined through the iterative engineering approach termed the Learn-Design-Build-Test (LDBT) cycle. This entails deriving insights from models and studies, devising genetic modifications, creating the engineered strain, and evaluating it in vitro and in vivo. Cyclic techniques, combined with high-throughput screening (e.g., concurrently evaluating numerous enzyme variants or circuit designs), expedite the optimization of engineered microorganisms (Liao et al. 2022).

The current toolkit for gut microbe engineering includes: metabolic modeling frameworks for predicting engineering outcomes; CRISPR-based genome editing applicable to various species; synthetic promoters and gene circuits tailored to gut environments; advanced protein secretion systems; and the iterative computational redesign of enzymes. These advancements collaboratively enhance the consistency and systematic nature of therapeutic microbe generation more than ever before. Utilizing enzymes and circuits optimized for the human gastrointestinal environment, meticulous microbial engineering will assist in tackling complex disorders. The key components of this modern engineering toolkit, including editing tools, circuit systems, secretion modules, and modeling platforms are summarized in Table 1 to provide a consolidated reference for current capabilities in gut microbe engineering.

**Table 1:** Genetic engineering tools and synthetic biology elements for gut microbe engineering

Tool/element	Representative studies/applications	Key features	Microbial hosts	References
Plasmids & vectors	Broad-host-range plasmid replicons (e.g. pWV01, pSH71, pAMβ1) enable gene expression in diverse LAB and *E. coli*. *E. coli*–*Bifidobacterium* and *E. coli*–*Bacteroides* shuttle vectors have been constructed for genetic transfer.	Allow stable maintenance of introduced DNA either episomally (with suitable ori and selectable markers) or by chromosomal integration. Overcoming host restriction barriers (e.g. via DNA methylation) dramatically improves transformation efficiency. Conjugation-based transfer and transposon delivery are also employed in situ.	Gram-positive probiotics (*Lactococcus*, *Lactobacillus* spp.), Actinobacteria (*Bifidobacteria*), Gram-negative commensals (*Bacteroides* spp.), model *E. coli* strains.	(Bober et al. 2018)
Promoters & regulators	Libraries of synthetic constitutive promoters/RBS variants achieve > 10^4^-fold expression range in *Lactobacillus plantarum* and *Bacteroides thetaiotaomicron*. Inducible systems adapted from native circuits: e.g. nisin-inducible (NICE) in *L. lactis* and sakacin-P pheromone system in *L. sakei*; chemical inducers like IPTG or xylose function in *L. plantarum* and *B. thetaiotaomicron*. Quorum-sensing promoters and two-component regulators enable responsive control in gut conditions.	Synthetic promoters provide tunable gene expression ranges; inducible promoters allow exogenous control of gene circuits. Quorum and two-component systems act as bioswitches responsive to microbial or environmental signals. Must be optimized per host (e.g. sakacin system incompatible with *L. lactis*, while NICE is broad-host ).	Lactic acid bacteria (*Lactococcus*, *Lactobacillus*), *Bacteroides*, *E. coli*, and other engineered commensals.	(Bober et al. 2018)
Biosensors	Engineered *E. coli* Nissle detecting gut inflammation via two-component systems: a thiosulfate sensor from *Salmonella* and a tetrathionate sensor from *Shewanella* were integrated to report colitis in mice. Similarly, *E. coli* with a nitrate-sensing circuit was designed for real-time diagnosis of intestinal inflammation. Other biosensors sense pH, metabolites or pathogen signals and trigger reporters (e.g. luminescence, color) for diagnostics.	Synthetic biosensors couple environmental inputs to gene expression outputs. Typically built from natural promoters or two-component systems responsive to inflammation-associated molecules (e.g. tetrathionate, nitrate). Can be linked to reporters (fluorescent proteins, enzymes) for noninvasive readouts. Key design challenges include sensitivity within gut conditions and minimizing circuit burden.	Commonly *E. coli* (probiotic strains like Nissle 1917) due to available tools. Also implemented in other commensals or probiotics with known input-responsive promoters (some in *Lactococcus*, *Bacteroides*).	(Bober et al. 2018; Ngashangva and Chattopadhyay 2023)
Memory circuits	Recombinase-based genetic switches and CRISPR-based recorders enable gut bacteria to remember past events. For example, engineered *E. coli* with Record-seq (CRISPR spacer acquisition from RNA) recorded transcriptional responses during transit through the mouse gut under different diets and inflammation states. CRISPR spacer acquisition has also been used to create “memory arrays” capturing exposure to specific DNA or stress signals in vivo. Synthetic toggle switches (e.g. logic gates that flip states in response to stimuli) have been demonstrated in model strains (EcN) as proof-of-concept for in vivo memory.	Long-term genetic memory is achieved by permanent DNA changes (integration of spacers or inversion of segments), allowing bacteria to record environmental stimuli. Reversible circuits (toggles) use serine recombinases or repressors to store binary states. These circuits function as “data loggers” inside the gut, surviving cell divisions and enabling later readout. Key features include stability of the memory element and minimal fitness cost.	Feasible in engineered *E. coli* (well-characterized CRISPR and recombinase systems). Efforts are underway to port memory circuits into other gut commensals, though *E. coli* remains the primary chassis for in vivo memory studies.	(Bober et al. 2018; Drummond et al. 2022; Munck et al. 2020)
Protein secretion systems	Sec-pathway signal peptides and surface display systems are used to deliver therapeutic proteins at mucosal sites. For instance, *L. lactis* was engineered to secrete human IL-10 (using a leader peptide) to treat colitis. This strain (LL-Thy12) successfully delivered IL-10 locally in the gut and improved inflammation in a murine IBD model. Other examples include probiotics secreting cytokines (IL-2, IL-22) or antibodies to modulate gut immunity in vivo (preclinical studies). Type III secretion systems (from *E. coli* or *Salmonella*) have also been repurposed to inject effector proteins into host cells for therapeutic aims (e.g. antigen delivery for vaccines).	Signal peptide fusions target proteins to the secretion pathway, releasing bioactive molecules into the gut lumen or mucosa. Surface display techniques tether peptides/proteins on the bacterial surface (e.g. anchored enzymes or antigens). These systems enable direct host-microbe interaction, such as delivering cytokines, enzymes, or antigens at the site of disease. Key considerations are protein folding, activity after secretion, and containment of the transgene (often using auxotrophic strains for safety).	Lactic acid bacteria (e.g. *Lactococcus lactis*, *Lactobacillus* spp.) for cytokine/enzyme delivery; also *E. coli* and attenuated Salmonella for antigen or drug delivery in cancer therapy (investigational).	(Bober et al. 2018; Braat et al. 2006)
CRISPR genome editing	CRISPR/Cas9-based tools have enabled precise genome modifications in gut commensals. For example, Cas9 can be used as a counter-selection to facilitate allelic replacement in *Lactobacillus* and *Bacteroides* (only clones with successful homologous recombination survive Cas9 cleavage of wild-type DNA). CRISPR interference (CRISPRi) systems, using dCas9 to repress target genes, have been adapted on broad-host plasmids (e.g. Mobile-CRISPRi vectors) to knock down genes across diverse gut bacteria. Such tools allowed functional genomics in previously intractable species. Recently, CRISPR base-editors are being explored to introduce point mutations without double-strand breaks (in development).	Cas9-mediated editing provides a programmable “molecular scissor” for genome engineering in non-model microbes, dramatically increasing editing efficiency when paired with donor DNA templates. CRISPRi (using catalytically inactive Cas9) enables tunable gene silencing without permanent changes. These platforms accelerate strain engineering and pathway optimization in probiotic chassis. Key features include the need for efficient delivery (conjugative plasmids or phage) and avoiding host anti-CRISPR defenses.	Demonstrated in LAB (e.g. *Lactococcus*, *Lactobacillus*) and anaerobes like *Bacteroides*. Mobile CRISPRi has been applied to various gut commensals (including *E. coli* and diverse *Bacteroides*) by using broad-host conjugative plasmids.	(Bober et al. 2018; Peters et al. 2019)

## Next-generation genome editing and delivery technologies

Recent progress in CRISPR biology has added more tools for probiotic engineering than just the usual Cas9-based editing. Different Types of Cas9: New CRISPR nucleases like Cas12a (Cpf1) and Cas13 have their own unique benefits. Cas12a is a Type V enzyme that makes staggered DNA cuts and recognizes different PAM sequences that are rich in T residues. In human gut commensals like *Bacteroides*, an inducible FnCas12a system made very efficient genome edits without markers. These edits included large deletions (up to 48 kb) and targeted insertions, with overall editing rates often over 60% (even reaching 100% in *B. vulgatus*) (Wang et al. 2024a, b). At the same time, Cas13 (Type VI) works as an RNA-guided RNase, which means it can knock down specific genes at the RNA level without changing the genome. Researchers have changed Cas13 effectors in bacteria so that they can break down certain transcripts. This gives them a way to turn off genes or fight RNA-based pathogens in the same place (Liu et al. 2023). These Cas9 alternatives make probiotic engineering more useful by letting you edit AT-rich genomes and temporarily turn off gene functions.

Base Editing and Prime Editing: Instead of making double-strand breaks, CRISPR-based base editors and prime editors let you make exact point mutations and small changes to genes. Base editors are fusion enzymes, usually a deaminase linked to a nuclease-inactive or nicking Cas, that change one base into another (for example, C·G to T·A) right in the target sequence (Liu et al. 2023). Wang et al. (2024a, b) made a cytidine base editor (pnCasBS-CBE) in *Bacteroides thetaiotaomicron* that added stop codons or missense mutations to metabolic genes with efficiencies that ranged from about 15–100%. Researchers have successfully used these CRISPR base editing tools on a number of bacteria to turn off genes without relying on host repair pathways (Brödel et al. 2024). Prime editing takes this accuracy even further by using a Cas9 nickase fused to a reverse transcriptase and a special guide (pegRNA) to “search and replace” DNA sequences. A recent study on the probiotic *E. coli* Nissle 1917 showed how powerful prime editing is by making targeted base substitutions, insertions, and deletions in one step, with editing efficiency ranging from 50 to 67% depending on the change (Chen et al. 2025). This prime editing method caused fewer off-target mutations than regular base editors, and it let researchers add a unique DNA barcode to the genome without using a selection marker (Chen et al. 2025). So, with the help of base and prime editors, probiotic engineers can now make point mutations or add new genetic elements with very little damage to the rest of the genome.

New ways to deliver things: One of the biggest problems in engineering gut microbes is getting genetic tools into different types of bacteria that don’t always change. Bacteriophages have become powerful tools for delivering genes in living organisms. In the complicated gut environment, phage-based delivery gets around the need for conjugative donor strains or electroporation. Recent discoveries show that engineered phage particles can carry CRISPR parts directly to the microbes in the gut that they want to attack. For example, Brödel et al. (2024) made a non-replicating M13-derived phage capsule with a base editor inside it. This capsule was able to successfully edit a β-lactamase gene in an *E. coli* strain that was living in the mouse intestine. A single oral dose of this phage-delivered editor caused a desired point mutation in about 93% of the bacterial population, and the edited strain stayed in the gut for more than six weeks (Brödel et al. 2024). The phage vector was carefully designed not to spread, so the CRISPR payload wouldn’t spread beyond the target microbes. In addition to this study, other research has used lytic or filamentous phages to deliver CRISPR–Cas plasmids that either knock out genes or selectively kill antibiotic-resistant strains. This shows how flexible phage-assisted delivery can be. This phage-based method, along with older methods like conjugative plasmid transfer and encapsulated nanoparticles, is making it easier to change the genes of gut bacteria in place. Researchers can now make precise changes to the genomes of gut probiotic strains with unprecedented efficiency inside the living host by combining advanced editing techniques (base/prime editing) with targeted delivery methods (phage vectors). These technologies work together to make sure that engineered probiotics stay up to date with the most recent genome editing breakthroughs. This opens the door to more effective and predictable therapeutic microbes.

## Applications in microbial production systems for gut health

The confluence of mechanistic and technological advances has led to an explosion of applications where engineered microbes act as production systems in the gut, synthesizing beneficial compounds, degrading harmful ones, or modulating local physiological conditions. We discuss several major target areas below, illustrating each with recent high-impact examples.

### Inflammatory bowel disease and immune modulation

Chronic intestinal inflammation and dysregulated immune responses characterize inflammatory bowel disease (IBD), which encompasses Crohn’s disease and ulcerative colitis. Engineered microbes have been studied as a means to deliver anti-inflammatory compounds or activate immune-regulatory pathways directly at the site of inflammation, given the influence of the gut microbiome on mucosal immunity. One strategy involves enabling microbes to secrete immunomodulatory proteins or anti-inflammatory cytokines. The proof-of-concept *L. lactis* strain that secretes IL-10 (Steidler et al. 2000) substantially reduced colitis in mice and inspired numerous subsequent projects. Praveschotinunt et al. (2019) engineered *E. coli* Nissle to secrete biologically active anti-TNF nanobodies tethered to the cell surface via a matrix-binding domain. In an IBD model, this tailored anti-TNF administration mitigated inflammatory signals in the colonic mucosa, hence reducing disease severity (Ma et al. 2022). Wang et al. (2021) developed *E. coli* Nissle to secrete a parasitic helminth protein (Sj16) that induces regulatory immune cells; oral administration of this strain ameliorated colitis in mice by reinstating butyrate-producing commensals and elevating retinoic acid, thereby demonstrating a microbe-mediated rebalancing of the gut immune milieu.

Inflammatory bowel disease (IBD) is being studied through the use of specific synthetic consortia of created bacteria, rather than individual strains. Tanoue et al. (2019) identified a mix of 11 commensal strains that activate colonic regulatory T-cells; despite the absence of genetic modification, this research provides a framework for developing multi-strain therapies. In a two-strain system that utilizes a division-of-labor strategy, Riglar et al. (2017) introduced one *E. coli* strain that secretes an anti-inflammatory effector and another strain that monitors inflammation as a live diagnostic. Engineered consortia can now synchronize activities through the programming of interspecies communication, namely utilizing quorum sensing signals. One strain may identify an inflammatory marker and alert a second strain to administer a medicine, for instance. Although still nascent, this community-level engineering demonstrates potential to emulate the robustness of entire microbiota while maintaining the regulation of artificial systems.

### Infectious disease and pathogen defense

The concept of utilizing probiotics to fight pathogens is not new, but synthetic biology has made it possible to develop much more potent and targeted antibacterial methods (e.g., employing Lactobacillus to inhibit gut pathogens). Hwang et al. (2017) provided a notable example by altering *E. coli* Nissle to specifically target *Pseudomonas aeruginosa*, an opportunistic pathogen that may proliferate in the gut after antibiotic therapy. The modified *E. coli* was equipped with a genetic circuit that identifies *P. aeruginosa* quorum signals and subsequently produces a tailored bacteriocin and lytic peptides to eliminate Pseudomonas. This synthetic probiotic eliminated an existing *P. aeruginosa* infection in mice and prevented its formation when administered prophylactically (Yeh et al. 2024). Published in Nature Communications, this study demonstrated the potential of programmed intervention within the gut microbiome: a benign bacterium can operate as an intelligent “assassin,” activating solely in the presence of a specific disease.

Additionally, various tailored probiotics have been designed to combat infectious illnesses. Palmer et al. (2019) engineered *E. coli* Nissle to produce microcin H47, a narrow-spectrum antibiotic peptide, in response to tetrathionate, a biomarker for Salmonella infection. In a murine model of enteric infection, this strain selectively eradicated Salmonella, significantly reducing pathogen load (Yeh et al. 2024). The circuit ensures the timely and locationally appropriate production of microcin, preserving the normal microbiota, as tetrathionate levels increase during Salmonella-induced inflammation. A genetically modified yeast strain was developed to detect and bind *Clostridioides difficile* toxins in the colon, thereby mitigating their effects (Chen et al. 2020). These occurrences highlight a theme: biosensing-driven antimicrobial delivery, in which engineered microbes remain dormant in the gut until the presence of a pathogen or its virulence factor activates their defense mechanisms. In parallel with engineered probiotics, novel antimicrobial approaches such as silver nanoparticle therapy have shown efficacy against pathogens like Mycobacterium tuberculosis (Sur et al. 2022), suggesting complementary strategies to combat infections.

Certain modified strains have progressed to clinical trials for infectious purposes, which is significant. An exemplary case involves a synthetic biology company developing a Bacillus strain designed to harbor genes that decompose *C. difficile* toxin and reinstate colonization resistance; this product (VE303) constitutes a defined consortium aimed at preventing C. diff relapse. Although it is not genetically modified, it underscores the growing interest in microbiome-based interventions for infection. Academic laboratories are simultaneously engineering *E. coli* Nissle to eliminate specific pathogens such as Enterococcus faecium and Staphylococcus aureus (Drolia et al. 2020 altered Nissle to target Listeria in the gastrointestinal tract and safeguard against systemic infection). The application of targeted live biotherapeutics that specifically eliminate antibiotic-resistant or detrimental bacteria while maintaining the integrity of the remaining microbiota may reduce dependence on broad-spectrum antibiotics. Addressing multi-drug-resistant bacteria remains crucial; for instance, exposure to tellurite was found to inhibit an XDR strain of *Mycobacterium tuberculosis* and alter its efflux pump activity (Parveen et al. 2023), providing insight into tackling resilient infections.

### Metabolic disorders and chemical imbalances

Among the most clinically advanced applications of altered gut bacteria might be metabolic disorders, which are characterized by the construction of toxic metabolites or the absence of helpful ones. We already discussed PKU and hyperammonemia as exemplary cases: Phase 1 studies have been conducted for these conditions. Hyperammonemia was the inaugural human trial of an engineered probiotic; *E. coli* SYNB1020 was evaluated in cirrhotic patients but was discontinued in Phase 1/2 due to insufficient colonization. SYNB1618 for PKU has completed a Phase 1/2a trial demonstrating safety and dose-dependent phenylalanine metabolism to a benign biomarker in both healthy volunteers and patients. An enhanced strain (SYNB1934) exhibiting increased efficacy is presently undergoing Phase 2 studies. Published in Nature Metabolism, these clinical trials signify a pivotal moment as they confirm that engineered gut bacteria can be administered safely to humans and can execute a metabolic function—specifically, the consumption of an amino acid—within the complex environment of the human gastrointestinal tract (Adolfsen et al. 2021; Ma et al. 2022).

In addition to these, other metabolic objectives pursued include enteric hyperoxaluria, a condition characterized by excessive oxalate leading to kidney stones. Synlogic and Novome have developed intestinal microorganisms to metabolize dietary oxalate. Synlogic’s strain SYNB8802, engineered to metabolize oxalate, is an *E. coli* now undergoing Phase 1 trials (Lubkowicz et al. 2022a). Novome’s Phase 1/2a product is distinct; it is designed as a commensal *Bacteroides* (strain Nissle NB1000) incorporating an oxalate-degrading pathway and a dependency circuit, enabling stable engraftment in the gut upon administration of a particular polysaccharide (Ma et al. 2022). These endeavors demonstrate that designed biocatalysis in the gut can address systemic metabolic disorders: by degrading a molecule in the gut prior to its entry into circulation, the altered microbe serves as a living metabolic filter or organ.

The regulation of nutrition acquisition and obesity constitutes an emerging domain of study. To improve metabolic syndrome, Ma et al. (2022) engineered *E. coli* Nissle to secrete a glucagon-like peptide 1 (GLP-1) mimic. This strain resulted in reduced weight gain and improved glucose tolerance in obese mice, indicating its potential application as an anti-obesity probiotic. Although the process is somewhat hormonal, GLP-1 affects insulin secretion and appetite, this scenario demonstrates the utilization of gut microbiota to provide peptide therapeutics typically delivered via injection. Engineered bacteria have been developed to consume excess choline or trimethylamine in the gut, thereby reducing trimethylamine-N-oxide (TMAO), a molecule associated with heart disease (a hypothesis proposed by Romano et al. 2015). Despite remaining at the preclinical stage, these solutions exhibit significant potential: any metabolite present in the gut may, theoretically, be modified by a well-engineered microbe, thereby converting the gut into a viable site for therapeutic biochemistry.

### Gut-Brain axis and other emerging areas

Engineered bacteria producing neuroactive compounds have been facilitated by the influence of the gut microbiome on the nervous system i.e. the gut-brain axis. Certain gut bacteria metabolize dietary tryptophan into compounds that affect serotonergic transmission. Researchers are investigating the engineering of commensals to enhance the synthesis of GABA or serotonin precursors in the stomach, perhaps alleviating anxiety or unhappiness. A recent proof-of-concept (Sharma et al. 2024) developed *E. coli* to synthesize kynurenine derivatives that activate vagus nerve circuits, resulting in behavioral changes in mice. Despite being in its early stages, these investigations demonstrate that biocatalysis by gut bacteria may produce neuromodulators or neurotransmitters, effectively acting as miniature drug factories within the intestines that influence the brain.

The application of modified microbes in cancer therapy beyond the colon is a noteworthy area of research. Certain bacteria spontaneously localize to cancerous locations; for instance, certain Clostridia thrive in hypoxic tumor cores. Motivated by this, (Chowdhury et al. 2019) and others have engineered E. coli to selectively thrive in tumors and administer therapies such as immunomodulatory proteins. While similar methodologies are being explored in melanoma and liver cancer models, researchers in 2024 employed an altered E. coli Nissle for colon cancers. One design involves engineering bacteria to produce a prodrug-converting enzyme exclusively within tumors, so transforming a systemically administered inert drug into an active chemotherapeutic agent solely at the tumor site (termed “bacterial gene-directed enzyme prodrug therapy”). This mitigates systemic toxicity through precise in situ biocatalysis (Wang et al. 2024a, b). This emerging strategy, along with other engineered microbial therapies targeting metabolic and immune disorders, is systematically outlined in Table 2, which summarizes key clinical trials of engineered live bacterial therapeutics from 2000 to 2024.

The utilization of modified microbes as vaccines or vaccine adjuvants is on the rise. An example is a probiotic engineered to display pathogen antigens on its surface or produce them, so enhancing mucosal immunity. An engineered Lactococcus producing an HIV antigen has commenced preliminary studies to assess its safety in eliciting an immunological response (Bioengineered Lactococcus for oral vaccination, 2019). While it pertains more to antigen presentation than metabolic engineering, it nonetheless falls under the domain of manipulating gut microbes for health purposes. Live vaccine strains can be engineered with metabolic traits to enhance their viability until they deliver the antigen, integrating metabolic and immunological engineering (Faghihkhorasani et al. 2023). While these approaches are still evolving, Table 3 presents a broader range of engineered probiotic applications across disease contexts, showcasing the diversity of therapeutic mechanisms and microbial chassis used.

The applications of engineered gut microbial systems are extensive, encompassing the digestion of excess substrates (such as amino acids, ammonia, and oxalate), the production of deficient molecules (including short-chain fatty acids and hormones), the mitigation of infections, the regulation of immunity, and the influence on distant organs through metabolite signaling. Every application, whether involving an enzyme pathway or a regulatory circuit, relies on a meticulously engineered biocatalytic activity specifically intended for a certain therapeutic goal (Liu et al. 2022). Table 1 presents a non-exhaustive compilation of significant engineered probiotic strains along with their intended applications, illustrating the diversity of diseases addressed.

**Table 2:** Clinical trials of engineered live bacterial therapeutics (2000–2024)

Strain (engineer)	Engineered function	Target indication	Phase (year)	Sponsor/company	Outcome summary
*Lactococcus lactis* “LL-Thy12” (Genetically modified to secrete human IL-10)	Mucosal delivery of anti-inflammatory IL-10 (with thymidine auxotrophy for containment)	Crohn’s disease (IBD)	Phase I (2006) (Braat et al. 2006)	Academic/ActoGeniX	Safe and well-tolerated; minor adverse events only. Fecal recovery confirmed containment (requiring thymidine). Some reduction in disease activity observed, though in an open-label trial. Pioneering demonstration of a GM probiotic therapy in humans.
*Lactococcus lactis* “AG013” (Secreting human Trefoil Factor 1)	Secretion of Trefoil Factor-1 to promote oral mucosal healing	Oral mucositis in chemo/radiation patients (Head & Neck cancer)	Phase II (2020) (Ternyila 2020)	Oragenics/ActoBio	Safe oral rinse delivery; however, the trial missed its primary endpoint– no statistically significant reduction in severe oral mucositis duration vs. placebo. Therapy was well tolerated, but efficacy was inconclusive, leading to no approval from this study.
*Lactococcus lactis* “AG019” (Expressing human proinsulin and IL-10)	Local release of autoantigen (proinsulin) plus IL-10 to induce immune tolerance in the gut	Type 1 Diabetes (early-onset, immunotherapy)	Phase 1b/2a (2021–2023) (Mathieu et al. 2024)	Precigen ActoBio	Safe as oral capsule; no systemic exposure of strain or transgene products. Monotherapy stabilized C-peptide and glycemic control up to 6–12 months; in combination with an anti-CD3 antibody (teplizumab), patients showed preserved or improved β-cell function over 12 months. Preliminary data suggest enhanced tolerance induction, supporting further trials.
*E. coli Nissle* 1917 “SYNB1020” (Engineered urease cycle strain)	Ammonia assimilation to lower blood ammonia (engineered to consume ammonia and produce benign metabolites)	Hyperammonemia (e.g. Urea Cycle Disorder, hepatic encephalopathy)	Phase 1b/2a (2019) (Taylor 2019)	Synlogic, Inc.	Demonstrated safety and colonization in humans, but no efficacy in lowering blood ammonia compared to placebo. The strain did not significantly reduce plasma ammonia in cirrhosis patients, despite active biomarker signals, leading to termination of the program.
*E. coli Nissle* 1917 “SYNB1618” and improved variant “SYNB1934” (Engineered Phe-metabolizing strains)	Phenylalanine degradation via expressed phenylalanine ammonia lyase (PAL) to lower systemic Phe levels	Phenylketonuria (PKU, metabolic disorder)	Phase II (2023) (Synpheny-1 trial)(Vockley et al. 2023)	Synlogic, Inc.	In a 14-day study, both strains safely reduced blood Phe. SYNB1934 showed ~ 43% reduction in dietary Phe AUC and 40% drop in fasting Phe; SYNB1618 ~ 34% AUC reduction. No serious adverse events; some mild GI effects. Demonstrated proof-of-concept that synthetic biotics can lower an amino acid toxin in patients. Phase 3 planning underway.
*E. coli Nissle* 1917 “SYNB8802” (Engineered oxalate degrader)	Oxalate degradation in the gut to reduce urinary oxalate (expresses oxalate decarboxylase and formate metabolism)	Enteric hyperoxaluria (kidney stone prevention)	Phase 1 (2021–2022) (Lubkowicz et al. 2022b)	Synlogic, Inc.	Preclinical models predicted clinically meaningful reduction in urinary oxalate. A Phase 1 trial in healthy volunteers showed safety and on-target activity; subsequent pilot in patients showed ~ 38% decrease in urinary oxalate with the probiotic vs. placebo (company communication). Progressing to expanded trials after achieving proof-of-mechanism (reduced oxalate excretion).
*Bacteroides ovatus* “NOV-001 (NB1000)** (Engineered commensal with diet-dependent growth)	Colonization of gut and consumption of oxalate; strain requires a provided polysaccharide (NB2000 prebiotic) for niche control	Enteric hyperoxaluria (post-gastric bypass patients)	Phase 1 (2021) (Novome Biotechnologies 2021); Phase 2a (2022–24)	Novome Biotechnologies	First-in-human trial of a Genetically Engineered Microbial Medicine (GEMM). Phase 1 showed the engineered *Bacteroides* safely engrafts in the human gut with controlled density via the prebiotic “fuel”. No significant adverse effects. Phase 2a ongoing to assess efficacy in lowering urinary oxalate; early results indicate successful colonization and oxalate degradation in patients (company reports).
*Additional IND-Enabling Strains* (preclinical)	*Examples*: Engineered *E. coli* delivering immunomodulators (e.g. IL-22 or anti-inflammatory enzymes) for Ulcerative Colitis; Attenuated Salmonella carrying tumoricidal genes for cancer therapy. (Not yet in human trials as of 2024.)	Various (Inflammatory bowel disease, cancer, etc.)	Preclinical (IND-enabling) (Hamade et al. 2022, 2024)	Academic and biotech (e.g. Synlogic, Elypta, etc.)	A number of next-generation engineered probiotics are in late-preclinical stages. For IBD, strains secreting cytokines (IL-22, IL-10) or reactive oxygen species scavengers have shown disease attenuation in animal models. In oncology, bacteria like *E. coli* or *Salmonella* are being programmed to selectively deliver toxins or immune checkpoint nanobodies to tumor sites (promising in mice). These await clinical translation pending safety and regulatory approvals.

**Table 3:** Selected applications of engineered gut microbes (2000–2024)

Engineered microbe (chassis)	Therapeutic function	Target condition	Key innovation/outcome	References
*Lactococcus lactis* (N/A)	Secretion of IL-10 anti-inflammatory	Colitis (IBD)	Reduced intestinal inflammation in mice (first proof-of-concept for live microbial therapy)	(Steidler et al. 2000)
*E. coli* Nissle 1917 (EcN)	Thiosulfate sensor + reporter	IBD diagnosis	Detected gut inflammation via stool readings (memory circuit in bacteria)	(Daeffler et al. 2017)
*E. coli* Nissle 1917 (EcN)	Secretion of anti-TNF nanobody	Colitis (IBD)	Locally neutralized TNF, reducing inflammation in mouse colon	(Praveschotinunt et al. 2019)
*E. coli* Nissle 1917 (EcN)	Production of butyrate (5-gene pathway)	Metabolic syndrome	Increased butyrate levels modulated host endocannabinoid gene expression	(Hwang et al. 2017)
*E. coli* Nissle 1917 (EcN)	PAL + LAAD enzymes to degrade Phe	Phenylketonuria (PKU)	Lowered blood Phe ~ 38% in PKU mice; produced biomarker hippurate in primates	(Isabella et al. 2018)
*E. coli* Nissle 1917 (EcN), SYNB1020	Urease pathway for ammonia assimilation	Hyperammonemia	Reduced ammonia and increased survival in cirrhotic mice; Phase 1 trial (terminated)	(Kurtz et al. 2019)
*E. coli* Nissle 1917 (EcN)	Senses quorum & kills *P. aeruginosa*	Infection (PA gut colonization)	Cleared and prevented *Pseudomonas* gut infection in mice (conditional lysis and toxin production)	(Hwang et al. 2017)
*E. coli* Nissle 1917 (EcN)	Tetrathionate sensor ->Microcin H47	Infection (*Salmonella*)	Inhibited *Salmonella* growth in inflamed gut by on-demand microcin release	(Palmer et al. 2019)
*E. coli* Nissle 1917 (EcN)	Secretion of IL-22 & nanobodies (lysis in tumor)	Colorectal cancer (CRC)	Selectively colonized tumors; released immunotherapeutics (anti-PD-L1/CTLA-4, GM-CSF), reducing tumor burden ~ 50%	(Gurbatri et al. 2024)
*Bacteroides ovatus* (commensal)	Xylan-inducible KGF-2 delivery	Colitis (IBD)	Delivered growth factor to inflamed colon in mice, improving healing (using diet to trigger therapy)	(Hamady et al. 2010)
*Bacteroides thetaiotaomicron*	Responsive gene circuit (vitamin-induced)	Gut gene therapy (demo)	Tunable system for commensal gene expression in situ, demonstrated regulated enzyme delivery	(Lim et al. 2017)
*Lactococcus lactis* (AG013)	Secretion of Trefoil Factor 1 (human)	Oral mucositis (cancer therapy side effect)	Protected oral epithelium; tested in Phase 2 clinical trial (terminated)	Phase 2 trial data (Ma et al. 2022)
*Lactococcus lactis* (AG019)	Delivery of proinsulin and IL-10	Type 1 diabetes (T1D)	Aimed to induce immune tolerance to insulin; in Phase 1/2 trial for new-onset T1D	Phase 1/2 trial (Ma et al. 2022)
*Saccharomyces boulardii*	Engineered to bind C. diff toxin A & B	*C. difficile* infection	Neutralized toxins in gut, reducing disease severity in rodents (preclinical)	(Chen et al. 2020)
*Bifidobacterium longum* (bacTRL-IL12)	Secretion of IL-12 (cytokine)	Solid tumors (immunotherapy)	Being tested as oral immunotherapy to stimulate anti-tumor immunity (Phase 1 trial)	Phase 1 trial (Ma et al. 2022)

**Fig. 5:**
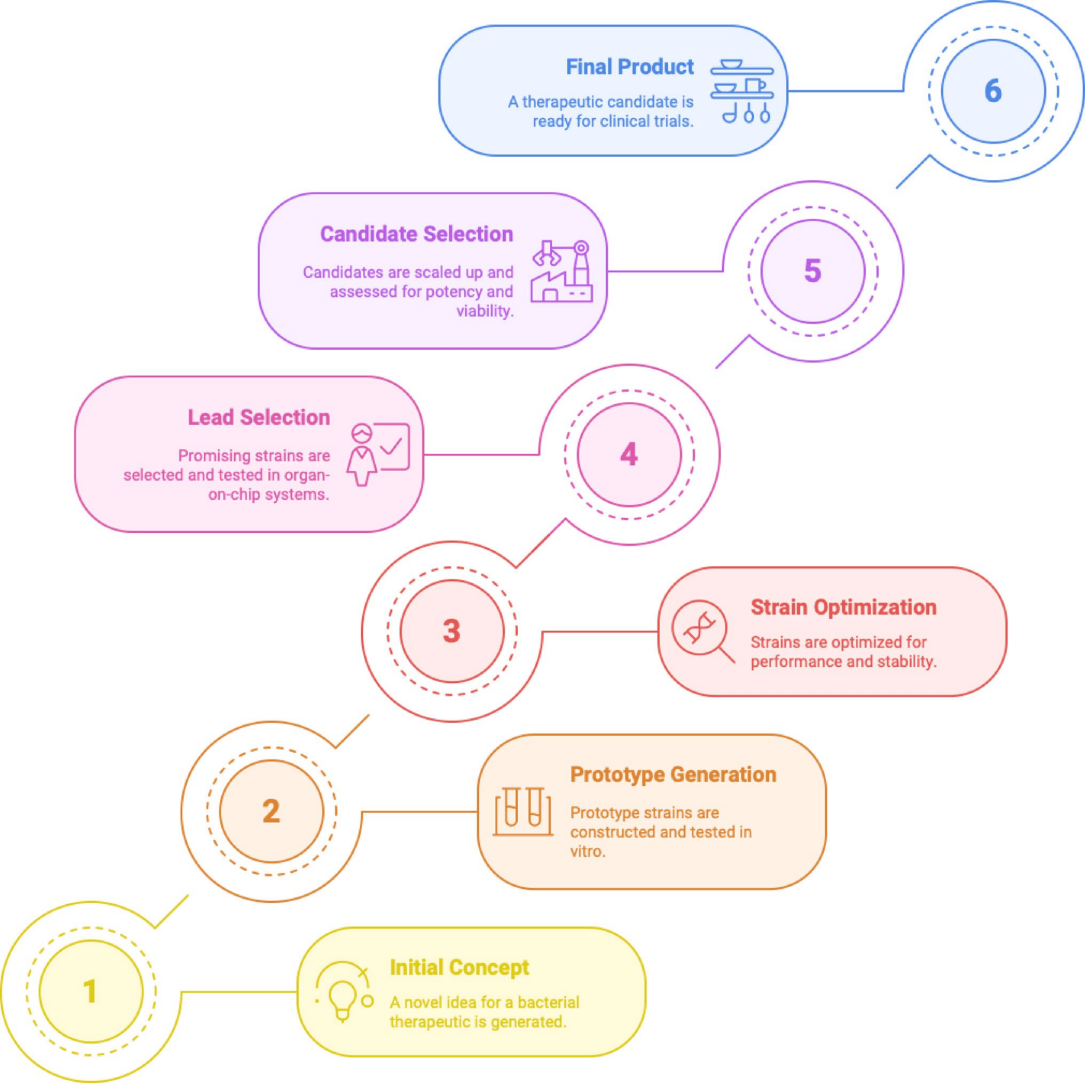
Stepwise development pipeline for engineered live biotherapeutics. This schematic outlines the six key stages in translating a live bacterial therapeutic from concept to clinical readiness. The process begins with Initial Concept (1), where a novel therapeutic idea is generated, followed by Prototype Generation (2), involving construction and in vitro testing of microbial strains. Strain Optimization (3) enhances performance, stability, and payload expression. In Lead Selection (4), promising candidates are screened through organ-on-chip platforms and other functional assays. Candidate Selection (5) focuses on scale-up, potency evaluation, and in vivo biomarker validation. The final stage, Final Product (6), represents a clinically viable live biotherapeutic candidate ready for human trials. This pipeline incorporates iterative feedback loops to ensure strain functionality, safety, and manufacturability at each step. (Adapted from Charbonneau et al. 2020)

The preceding examples and this table clearly demonstrate that modified microorganisms are being developed as multifunctional medicinal systems. They may function as living sensors and recorders, factories producing therapeutic molecules (e.g., cytokines, metabolites), or scavengers removing hazardous substances (toxins, inflammatory mediators), or perhaps embody all three roles. Significantly, some strains have transitioned from laboratory research to clinical application; several are currently in clinical development and highlighting that the field is evolving from theoretical concepts to practical implementation. The stepwise progression of these live biotherapeutic developments, from initial design through optimization and clinical validation which is captured in Fig. 5, which depicts the translational pipeline for engineered microbial therapeutics.

## Case study: *E. coli Nissle* 1917 as a chassis for therapeutic microbes

*Escherichia coli Nissle* 1917 (EcN) is frequently emphasized in the literature as a versatile platform for gut metabolic engineering. The non-pathogenic strain EcN, discovered over a century ago, is utilized as a probiotic in Europe for gastrointestinal disorders. Its extensive safety history in humans, ease of genetic manipulation (as an *E. coli*), and ability to inhabit the mammalian gut render it an attractive “chassis” for synthetic biology. EcN has thus been the focal point of numerous engineering initiatives across various disease domains. This case study illustrates the versatility and efficacy of metabolic engineering by emphasizing how a single strain can be reconfigured in various manners to tackle multiple gut-related challenges.

As previously stated, Synlogic Inc. has evolved EcN into the strain SYNB1618 for the treatment of phenylketonuria (PKU). This necessitated altering EcN with two significant enzyme pathways to enable the absorption of dietary phenylalanine in the gastrointestinal tract. However, despite promising early clinical data, Synlogic announced in February 2024 the discontinuation of its Phase 3 trial *Synpheny-3*, which evaluated **SYNB1934** (labafenogene marselecobac), an improved version of **SYNB1618** for treating phenylketonuria (PKU). The decision followed an internal review indicating the trial was unlikely to meet its primary endpoint, although safety was not a concern. Subsequently, the company initiated plans to cease operations and reduce its workforce by over 90%. While this outcome underscores the translational hurdles facing engineered therapeutics, such as microbial engraftment, inter-patient variability, and dosing complexity. But it does not undermine the potential of *E. coli* Nissle 1917 as a microbial chassis. SYNB1618, the precursor strain, had shown positive results in earlier clinical trials, validating both the concept and platform. Moving forward, these lessons highlight the need for improved pharmacokinetics, smarter synthetic designs, and perhaps personalized interventions to achieve clinical success.

Clinical investigations ensued from SYNB1618’s efficacy in preclinical models, which involved the reduction of blood phenylalanine and the generation of a quantifiable biomarker (trans-cinnamic acid converted to hippuric acid). Published in 2021 in Nature Metabolism, SYNB1618 demonstrated safety and tolerability in a first-in-human experiment, effectively metabolizing phenylalanine to hippurate in a dose-dependent manner in both healthy individuals and those with phenylketonuria (PKU). In light of continuous advancements in biocatalysis, an enhanced strain including ideal enzymes, SYNB1934, has progressed to Phase 2. The rational design of a metabolic pathway in EcN, animal validation, a biomarker method for monitoring (hippurate), and clinical translation (Charbonneau et al. 2020) collectively illustrate the comprehensive process in this case.

The heterologous urease and alanine dehydrogenase enzymes were employed to modify the EcN chassis, enabling it to address hyperammonemia by converting ammonia into alanine and other biomass constituents. SYNB1020 has progressed to human trials in individuals with cirrhosis. Despite the trial demonstrating no efficacy and the program being discontinued, it provided valuable data. The strain was found to transiently and in limited quantities colonize the human gut, indicating a necessity for improved engraftment or dosage methodologies (Ma et al. 2022). The engineered EcN demonstrated markedly superior ammonia clearance in mice and protected the subjects from ammonia-induced neurotoxicity (Kurtz et al. 2019). The acquired knowledge has informed contemporary designs; for instance, ensuring that an engineered strain possesses a metabolic advantage or specific niche (such as a unique nutrient it can exclusively utilize) for survival in the gut is now recognized as essential (Shepherd et al. 2018) engineered EcN to require a rare sugar, thereby allowing dietary provision of that sugar to selectively enhance the strain’s engraftment).

EcN has undergone multiple modifications for IBD. A compelling example is the development of EcN strains that respond to inflammation by releasing immunoregulatory compounds. Ameredes et al. (2005) developed an EcN that identifies nitric oxide, a hallmark of inflammation, and subsequently produces IL-10. An engineered microorganism designed to excessively manufacture short-chain fatty acids such as butyrate and propionate to enhance the mucosal barrier in colitis serves as another illustration. An altered EcN that produces butyrate by the incorporation of Clostridia genes was shown in a 2020 study to reduce inflammatory damage in a mouse colitis model, ostensibly by nourishing colonocytes and activating Treg cells; the precise mechanisms are currently under investigation. These efforts aim to transform EcN into a living anti-inflammatory agent: it discreetly monitors the stomach while releasing compounds to reestablish immunological equilibrium upon detecting inflammation (Bai and Mansell 2020). Although preliminary animal studies are promising, the modular architecture of EcN engineering facilitates the integration of multiple effectors; for instance, a future EcN could be engineered to concurrently release IL-22 (to enhance epithelial repair) and IL-10 (to mitigate inflammation), thereby providing a multifaceted therapeutic approach.

Hwang et al. (2017) engineered EcN to combat *P. aeruginosa*, as observed. EcN possesses inherent probiotic properties against specific infections, which engineers have augmented. To inhibit cholera infection by disrupting the quorum-sensing signals of Vibrio cholerae, a research group introduced a gene into EcN to interfere with pathogen communication, so diminishing pathogen virulence without eliminating it. A different group modified EcN to express a surface antigen that binds Helicobacter pylori, hypothesizing that EcN in the stomach could sequester H. pylori and reduce its colonization, a strategy currently under investigation. These endeavors share a commonality in employing EcN as a versatile delivery mechanism, capable of being equipped with antibacterial agents, competitive exclusion tactics, or vaccine antigens.

One of the most advanced applications of EcN to date is in the treatment and diagnosis of cancer. In a 2015 study, researchers led by (Danino et al. (2015) modified EcN to perform a three-fold role in colorectal cancer (CRC): targeting, sensing, and treating. Initially, they shown that when administered orally to mice, EcN inherently accumulates in tumors, especially in inflammatory or necrotic regions. They engineered EcN with a genetic circuit capable of lysing and releasing therapeutic agents upon sensing the tumor microenvironment: specifically, the cytokine GM-CSF to elicit an immune response, and checkpoint inhibitor nanobodies targeting PD-L1 and CTLA-4 to locally inhibit the immunosuppressive signals of cancer. The engineered strain produced a small chemical (salicylate) detectable in urine, serving as a diagnostic indicator for early tumor identification. The oral injection of this modified EcN in mice led to selective colonization of colorectal tumors and around a 50% reduction in tumor burden, along with the identification of a “signal” in the urine of tumor-bearing animals, which was absent in healthy ones. Integrating many designed functionalities onto a single chassis organism converts this remarkable case study into a theranostic agent, serving both diagnostic and therapeutic purposes. EcN is a unique platform for complex tasks due to its inherent tumor-homing ability coupled with synthetic circuits.

Researchers have engineered EcN with safety “kill switches” to prevent inadvertent persistence or dissemination across many applications. Numerous engineered EcN strains, for instance, possess auxotrophic mutations, indicating they require a nutrient absent from the human diet, thus necessitating supplementation for survival, hence ensuring biocontainment (Riglar and Silver 2018). Certain genes are inducible suicide genes that can be activated to eliminate the strain when necessary. Clinical experiments involving EcN-based modified strains have thus far demonstrated no significant adverse effects, reinforcing the notion that EcN can be safely employed as a therapeutic chassis.

Ultimately, *E. coli* Nissle 1917 demonstrates how a singular probiotic strain can be re-engineered for many therapeutic applications by integrating distinct biocatalytic modules and genetic circuits. EcN has served as the foundation for numerous pioneering demonstrations of tailored microbial therapy, addressing metabolic diseases, infections, and cancer. The transition from a World War I-era probiotic to a 21st-century synthetic biology platform exemplifies the evolution of the sector. EcN’s insights are currently informing the engineering of additional gut bacteria, including anaerobes such as *Bacteroides*, thereby expanding the repertoire of available chassis organisms.

## Determinants of success and failure in engineered gut microbial therapies

There are many promising examples of engineered microbes, but there are a few important things that need to happen before these treatments can be used in real life. Notably, many early trials have not been able to show that engineered LBP works in humans as well as it does in animals. About half of the terminated trials said that the results were not meaningful (Rutter et al. 2022).

Key factors includes, firstly, strain viability and persistence: The most important thing is whether the introduced microbe can survive and work in the gut. Engineered strains don’t usually colonize well; for instance, an *E. coli* Nissle chassis stayed in humans for an average of 48 h (Rutter et al. 2022). SYNB1618 for PKU was safe, but it was gone from the GI tract about four days after the last dose (Vockley et al. 2023). This temporary persistence can be good for safety and controllable pharmacokinetics, but it means that the drug needs to be given again and again to keep working. The strain’s ability to survive in the GI tract (exposed to stomach acid, bile, changing pH, etc.) can limit its effectiveness. In some cases, trials have failed because the gut bacteria didn’t survive or didn’t attach properly. For example, Synlogic’s ammonia-consuming strain (SYNB1020) improved hyperammonemia in animal models but didn’t lower ammonia levels significantly in cirrhotic patients, which led to the end of a Phase 1b/2a trial. Researchers are working on ways to make strains more robust without putting safety at risk (Rutter et al. 2022). These include acid-resistant capsules, biofilm-forming strains, and auxotrophic safety circuits.

Secondly, dosing strategies and pharmacokinetics: Engineered microbial therapies make it hard to tell the difference between a drug and a probiotic, so dosing must be done very carefully. Because most strains don’t stay in place permanently, the therapeutic effect is closely tied to how often and how much is given. For instance, the PKU treatment SYNB1618 made more Phe metabolites when given in higher doses and was given up to three times a day to keep working (Vockley et al. 2023). To get enough time in the gut, people often need to take high doses more than once (Rutter et al. 2022). On the other hand, some newer methods try to achieve long-term colonization with shorter dosing courses. For example, the defined *C. difficile* consortium VE303 achieved stable engraftment for up to a year when given for several days after an antibiotic pretreatment to “open” a niche (Dsouza et al. 2022). When thinking about pharmacokinetics, you need to think about how quickly the bacteria move through the body or are removed, whether they stick to mucosal surfaces, and whether their metabolic output can reach therapeutic levels before washout. One way to improve microbial pharmacokinetics is to time doses with meals or give prebiotics that help the engineered strain at the same time. In short, when designing dosing regimens, you have to find a balance between safety and persistence, like using non-colonizing strains for predictable clearance versus encouraging engraftment for long-term delivery (Rutter et al. 2022).

Thirdly, interactions between the host and the microbiome: the gut ecosystem and the patient’s health have a big effect on the results. Native microbiota can either compete with the therapeutic strain or break down its products, which makes them less effective. The host’s disease state, diet, immune responses, and microbiome composition all affect how well and how much colonization occurs (Rouanet et al. 2020). For instance, an inflamed or dysbiotic intestine may not have the nutrients that the engineered microbe needs, or it may have microbes that fight against it. The engineered microbe’s performance can be affected by things like the rate at which the stomach empties, the pH of the intestines, the amount of oxygen, and the immune system’s ability to clear it (Charbonneau et al. 2021). In real life, antibiotics (to cut down on competition) or dietary supplements (to give specific nutrients) have been used to help with engraftment (like VE303’s vancomycin lead-in) (Dsouza et al. 2022). On the other hand, host immunity and safety rules often mean that strains have to be auxotrophic or biologically contained, which can make it harder for them to grow in vivo. The case of AG013 (*L. lactis* secreting trefoil factor for oral mucositis) shows how hard these problems can be. It was safe and showed local TFF1 delivery, but the complicated oral environment (saliva flow, microbiome, tissue damage) meant that it didn’t work any better than a placebo in Phase II (Rutter et al. 2022). This shows that even a well-designed microbe may not be able to have a therapeutic effect without a supportive niche.

In general, these things, how well the strain survives, how well it is dosed, and how it interacts with the host environment and decide whether an engineered microbe therapy works. Future work will focus on using predictive modeling and advanced in vitro simulations to figure out these problems ahead of time, as well as creating solutions (like synthetic consortia, biofilm-forming chassis, and responsive gene circuits) that work best in the changing conditions of the human gut. Researchers want to turn promising designs into reliable clinical outcomes by taking into account strain viability, pharmacokinetic behavior, and host-microbiome dynamics.

## Limitations and current challenges

Therapeutic engineering of gut microbes like *E. coli* Nissle 1917 (EcN) holds significant promise, but several key limitations remain. Traditional approaches like Fecal Microbiota Transplantation (FMT) and conventional probiotics suffer from a lack of precision, batch-to-batch variability, and poorly defined mechanisms of action, limiting reproducibility and scalability. These drawbacks have created the rationale for more controlled and tunable interventions via engineered microbes, where specific functions can be programmed for targeted therapy.

However, engineered strains also face critical challenges. One of the major issues is inconsistent microbial engraftment and persistence in the host gastrointestinal environment, which is shaped by factors such as diet, native microbiota composition, and immune responses that vary between individuals. This variability compromises reproducibility and therapeutic efficacy across diverse populations. Moreover, the pharmacokinetics of live biotherapeutic products are not yet well-defined, and factors like colonization dynamics, dosing, and microbial metabolism behave unpredictably in clinical settings.

Additionally, regulatory and ethical hurdles complicate clinical translation of genetically modified organisms (GMOs). Although safety mechanisms like kill switches and auxotrophy-based containment systems have been developed, their long-term validation and acceptance remain works in progress. Furthermore, our mechanistic understanding of host-microbe interactions, especially at systems biology and molecular levels, is still incomplete, posing challenges to treatment predictability.

While engineered gut microbes like *E. coli* Nissle 1917 have shown promising results in preclinical and early clinical studies, none have yet reached widespread clinical application, primarily due to unresolved challenges in safety, dosing standardization, and host-specific variability. This underscores the need for a cautious and evidence-driven approach in translating these advances to patient care.

These limitations emphasize the need for interdisciplinary efforts combining synthetic biology, computational modeling, and personalized design strategies to advance safe and effective microbial therapies.

## Future directions and therapeutic outlook

Recent advancements in engineering gut flora indicate the emergence of a novel class of “living medicines.” A multitude of critical challenges and inquiries will shape the future trajectory of this discipline as it transitions from discrete academic demonstrations to broader clinical applications. The regulatory authorities are actively framing LBPs, which poses safety issues. Engineered microbes must meet stringent safety standards demonstrating that they do not horizontally transfer genes, do not exhibit invasive behavior, and can be eradicated if necessary. Biocontainment, as a broader system-level safety strategy, encompasses multiple approaches to prevent engineered microbes from persisting or spreading outside the intended environment. Within this framework, “kill switches” represent specific genetic tools designed to trigger microbial self-destruction under defined conditions. Other biocontainment methods include auxotrophic dependencies and environmental sensors that limit microbial survival. These tools are increasingly seen as essential components for the clinical deployment of synthetic probiotics (Riglar and Silver 2018; Wegmann et al. 2017). Manufacturing processes must ensure the uniformity of genetically modified strains, which is complex due to the potential for bacterial evolution. These problems can be solved thanks to the successful Phase 1/2 studies that have been done in the last five years for PKU and other conditions. The EMA has provided guidance on environmental risk assessments for live GMOs used in therapy, while the FDA has classified many modified probiotic candidates as orphan medicines.

A noteworthy challenge is that modified strains may not persistently engraft in the patient’s gut, especially if the patient’s microbiome outcompetes them. Future endeavors are focused on facilitating engraftment, such as creating a distinct niche for the designed bacterium. Shepherd et al. (2018) propose a strategic method of engineering a strain to metabolize a specific carbohydrate that is inaccessible to other bacteria, thereafter incorporating that carbohydrate into the diet to confer a competitive advantage to the strain. Periodic re-dosing, essentially administering the modified microbe as a regular treatment rather than a singular remedy, is an alternative technique. The integration of modest microbiome conditioning, such as a short antibiotic pretreatment to create a niche, is also being evaluated to facilitate the establishment of imported strains. These strategies will become more accurate as our understanding of microbe-microbe and microbe-host interactions expands, ensuring that modified microorganisms consistently perform their intended function within the target population. Future designs may integrate many therapeutic activities to tackle complex diseases, despite current designed strains often serving a singular function. Addressing IBD may be enhanced by a strain that fortifies the epithelial barrier and promotes regulatory immune cells, alongside providing anti-inflammatory effects. Achieving this may involve utilizing a consortium in which each member fulfills a distinct purpose or integrating many gene circuits, each addressing a specific aspect, into a single strain. The design issue is to coordinate multi-strain consortia to ensure harmonious functionality and to prevent overburdening any single microorganism, which may diminish fitness. The continuous advancement of circuit design automation and computational modeling (e.g., Cello adapted for *Bacteroides* (Taketani et al. 2020; Whitaker et al. 2017)] will facilitate the construction of intricate therapeutic networks with reduced trial-and-error through circuit design automation.

An intriguing frontier involves tailoring synthetic bacteria to individual microbiomes or genetic configurations, essentially precision medicine with microbes. If baseline metabolite levels or niche availability fluctuate, the optimal designed strain for an individual with microbiome X may differ from that of an individual with microbiome Y. In the future, it may be possible to analyze a patient’s microbiome and select or engineer a microbial therapy tailored to that specific environment, such as choosing a chassis compatible with their microbiota makeup or modifying the strain’s food requirements to align with the patient’s diet. Moreover, certain individuals may require modifications to the surface antigens of the modified strain to mitigate immunogenicity, as their immune systems may exhibit varying responses to bacterial therapies. While this individualization enhances complexity, the modular nature of synthetic biology may provide rapid customization once a foundational platform strain (such as EcN or *Bacteroides*) is established. Preliminary signs of this tendency encompass efforts to modify gene expression levels in engineered strains based on a patient’s metabolite profile or phage-mediated delivery for the insertion of functional genes into a patient’s gut microbiota as an alternative to administering live strains.

Engineered microorganisms are likely to augment traditional therapies. In metabolic illnesses, they may serve as maintenance therapy subsequent to gene therapy or reduce the necessary dosage of small-molecule drugs. In inflammatory bowel disease (IBD), they may be utilized in conjunction with biologics, such as monoclonal antibodies, to achieve deeper remission; for instance, microbial therapy could maintain mucosal repair between anti-TNF medication infusions. Engineered EcN bacteria in cancer may be integrated with systemic immunotherapies to stimulate both local and systemic immune responses (Gurbatri et al. 2024). These combinatorial tactics may become standardized, and a crucial area of research will be the interactions between modified microorganisms and pharmaceuticals (pharmacokinetics/pharmacodynamics). Given these modified microbes reside in the gut, they may affect the absorption and metabolism of concurrently provided oral medications, an aspect to monitor attentively in trials.

The introduction of genetically modified bacteria into patients, and potentially into the environment through fecal shedding, presents ethical dilemmas. It is crucial to ensure that engineered traits do not inadvertently spread to other bacteria; hence, employing non-transmissible plasmids or chromosomal integration for critical genes and avoiding selection indicators such as antibiotic resistance genes whenever possible is advisable. Clear explanation of their benefits and safety will facilitate public acceptance of “GMO probiotics.” Long-term surveillance of patients treated with LBPs may be necessary to confirm that modified strains do not induce late effects or survive beyond the planned timeframe. The majority of modified strains developed to date are intended to be self-limiting, which is reassuring as many utilize food-grade organisms, so mitigating safety concerns. Collaborative efforts among scientists, physicians, and authorities will establish guidelines that effectively promote the advancement of these treatments.

The advancement in gut microbial engineering is indisputable. The domain has transitioned from anecdotal achievements to a continuum of treatment options, with some already administered to patients, between about 2018 and 2024. Every notable advancement in tools or knowledge, be it a new method for sustaining an engrafted strain, an innovative sensor circuit, or a more potent enzyme, contributes directly to enhancing the efficacy and reliability of these therapies. In the near future, physicians may administer designed probiotics similarly to antibiotics or biologic drugs, utilizing specific strains tailored to particular illnesses. Probiotics, even those that are a century old, can acquire new capabilities through metabolic engineering, evolving into advanced medicines that improve human health in ways that natural microorganisms cannot, as evidenced by our model *E. coli* Nissle (Charbonneau et al. 2020). Notably, the power of microbial engineering extends beyond gut therapeutics– microbes have even been engineered to synthesize useful nanomaterials from agricultural waste (Sadhu et al. 2024), underscoring the vast biotechnological potential of these biocatalytic systems.

Engineered microbes are also being deployed in environmental contexts; for example, specialized bacterial consortia can aid in efficient wastewater treatment to protect water resources (Das et al. 2022).The integration of biocatalysis and metabolic engineering with the diverse gut microbiome has established an entirely novel approach to medicine: living therapies operating biochemically within the human body. If the progress of the past five years is any indicator, the coming years will see this modality transition from experimental to mainstream, thereby providing innovative therapy for illnesses that were previously difficult to manage. The gut microbiota, once only observed, is now being actively manipulated; this significant fusion of engineering and biology holds the potential to revolutionize illness treatment at the fundamental, microscopic level.

## Data Availability

No datasets were generated or analysed during the current study.
